# Metaphor Is Between Metonymy and Homonymy: Evidence From Event-Related Potentials

**DOI:** 10.3389/fpsyg.2020.02113

**Published:** 2020-09-01

**Authors:** Anna Yurchenko, Anastasiya Lopukhina, Olga Dragoy

**Affiliations:** ^1^Center for Language and Brain, National Research University Higher School of Economics, Moscow, Russia; ^2^Epilepsy Center, Moscow, Russia; ^3^Vinogradov Institute of the Russian Language, Russian Academy of Sciences, Moscow, Russia; ^4^Federal Center for Cerebrovascular Pathology and Stroke, Pirogov Russian National Research Medical University, Moscow, Russia

**Keywords:** ambiguous words, polysemy, metonymy, metaphor, homonymy, event-related potentials, N400, P600

## Abstract

The goal of the present study was to investigate the interaction between different senses of polysemous nouns (metonymies and metaphors) and different meanings of homonyms using the method of event-related potentials (ERPs) and a priming paradigm. Participants read two-word phrases containing ambiguous words and made a sensicality judgment. Phrases with polysemes highlighted their literal sense and were preceded by primes with either the same or different – metonymic or metaphorical – sense. Similarly, phrases with homonyms were primed by phrases with a consistent or inconsistent meaning of the noun. The results demonstrated that polysemous phrases with literal senses preceded by metonymic primes did not differ in ERP responses from the control condition with the same literal primes. In contrast, processing phrases with the literal sense preceded by metaphorical primes resulted in N400 and P600 effects that might reflect a very limited priming effect. The priming effect observed between metonymic and literal senses supports the idea that these senses share a single representation in the mental lexicon. In contrast, the effects observed for polysemes with metaphorical primes characterize lexical access to the word’s target sense and competition between the two word senses. The processing of homonyms preceded by the prime with an inconsistent meaning, although it did not elicit an N400 effect, was accompanied by a P600 effect as compared to the control condition with a consistent meaning of the prime. We suppose that the absence of the N400 effect may result from inhibition of the target meaning by the inconsistent prime, whereas the P600 response might reflect processes of reanalysis, activation, and integration of the target meaning. Our results provide additional evidence for the difference in processing mechanisms between metonymies and metaphors that might have separate representations in the mental lexicon, although they are more related as compared to homonyms.

## Introduction

Theoretical studies on ambiguous words traditionally distinguish between homonymy and polysemy (Lyons, 1977; Cruse, 1986). In homonymy, a word accidentally carries two or more unrelated meanings, e.g., *bank 1* “a financial institution” and *bank 2* “an area of land along the side of a river”; while in polysemy, a word has several related senses, e.g., *funny rabbit* “a small animal” and *tasty rabbit* “the meat from a rabbit.” Within senses of polysemes, two types of relations with the original literal sense can be distinguished: metonymy and metaphor (Apresjan, 1974; Pustejovsky, 1995; Geeraerts, 2010). Metonymy is motivated by contiguity: the shift from the original sense to a metonymic sense occurs within the same semantic domain, e.g., *funny rabbit → tasty rabbit*, where the focus of attention shifts from the whole animal to its particular part – meat. Metonymic shifts are regular and predictable, following typical patterns (e.g., animal/food, capital/government, producer/product; Apresjan, 1995; Littlemore, 2015). Metaphor is motivated by analogy: one entity is presented in terms of another, e.g., *mouth of a child* “the part of the face” and *mouth of the cave* “the entrance to something” (Lakoff and Johnson, 1980). Metaphorical shifts are not always obvious to speakers (Apresjan, 1974) and may have little in common with literal senses from which they were derived, e.g., *polluted atmosphere → relaxed atmosphere*. Therefore, from the point of view of linguistic theory, literal and metaphorical senses are considered closer to homonyms than literal and metonymic senses (see Apresjan, 1974).

Experimental psycholinguistic studies show that relatedness of the senses/meanings affects comprehension of ambiguous words and results in processing differences between polysemes and homonyms. These differences are usually explained by the theoretical assumption that related senses of polysemes have a single semantically rich representation in the mental lexicon, which can facilitate their processing (Nunberg, 1979; Pustejovsky, 1995). In contrast, homonyms are suggested to have multiple unrelated meanings competing for activation during ambiguity resolution, which might slow down word recognition. The lexical decision study by Klepousniotou and Baum (2007) showed that both types of polysemes are processed in isolation faster and more accurately as compared to homonyms. Similarly, sentences associated with one of the word senses/meanings produce a more robust priming effect on metonymies and metaphors relative to homonyms (with ambiguous words that are semantically unrelated to the prime as a control condition; Klepousniotou, 2002). In addition, eye-tracking studies testing the processing of ambiguous words in a sentence context showed that homonyms are processed more slowly relative to polysemous and unambiguous words (Frazier and Rayner, 1990; Pacht and Rayner, 1993; Brocher et al., 2018).

Psycholinguistic studies also demonstrate a processing advantage for metonymies as compared to metaphors (Klepousniotou, 2002; Klepousniotou and Baum, 2007; Bambini et al., 2013). Klepousniotou (2002) indicates that metaphor is less systematic and constrained than metonymy, situated between metonymy and homonymy on the ambiguity continuum. Using a sensicality judgment task, Bambini et al. (2013) showed that metonymies are easier to process in a sentence context as compared to metaphors: while metonymies (e.g., *That student reads Camillieri*) did not differ significantly from the literal interpretation (*That reporter interviews Camillieri*) in accuracy or reaction times, metaphors (e.g., *Those dancers are butterflies*) were processed less accurately and slower relative to the literal condition (*Those insects are butterflies*). The authors relate these difficulties to a lower availability of metaphorical senses and additional processing load. They argue that these results are in line with the cognitive linguistics approach, according to which metonymy is based on a single cognitive domain, whereas metaphor reflects mapping between two distinct cognitive domains (Lakoff and Johnson, 1980; Lakoff and Turner, 2009). The dissociation between metonymies and metaphors was also observed in the semantic clustering study by Lopukhina et al. (2018): in a clustering experiment, participants were presented with short phrases containing a polysemous word in literal, metonymic, or metaphorical senses (e.g., *perelom pozvonka* “vertebral fracture,” *perelom cheshetsa* “the fracture itches,” *istoricheskij perelom* “historical crisis”) and were asked to sort them into virtual baskets, so that phrases with the same perceived sense were grouped together. Participants often confused literal senses with metonymic senses of the words, but not any other pairs of senses.

Klepousniotou et al. (2008) investigated the representation of ambiguous words in the mental lexicon based on the priming effect between senses/meanings of the same word. They analyzed the effect of consistency between the prime (e.g., *shredded paper* or *daily paper*) and target (*wrapping paper*) phrases on processing words with high (predominantly metonymies), moderate (predominantly metaphors), and low (predominantly homonyms) semantic overlap between senses or meanings in a sensicality judgment task for the two-word pairs presented visually. The difference in reaction times and accuracy between conditions with consistent and inconsistent primes was found to be more pronounced for phrases with metaphors and homonyms as compared to metonymies. The authors indicate that, in contrast to metaphors and homonyms, senses of metonymies have a single lexical representation that includes features associated with all senses or they share a “core meaning” tied to the dominant sense. In addition, the conflict between prime and target may be easier to resolve because of highly overlapping senses.

Neurolinguistic studies using the methods of event-related potentials (ERP) and magnetoencephalography (MEG), which both have a high temporal resolution, shed even more light on processing ambiguous words. Beretta et al. (2005) showed that the neuromagnetic response during a lexical decision task was modulated by the number of word senses and meanings. Similarly to reaction times, the latency of the M350 response (a neuromagnetic evoked response that peaks around 350 ms post-stimulus and is associated with lexical access; Embick et al., 2001; Pylkkänen et al., 2002) was earlier for words with many senses as compared to words with few senses. In contrast, both reaction times and M350 latency were longer for homonyms than for words with a single meaning. Thus, the MEG results were in line with behavioral results of this experiment, suggesting that different homonymous meanings may compete with each other, slow down the activation process, and involve processing mechanisms distinct from those involved for words with many senses.

To investigate processing and lexical representations of different senses/meanings of ambiguous words, a number of ERP studies analyzed the priming effect that ambiguous words without preceding context exert on targets that are either related or unrelated to their dominant or subordinate sense/meaning. Klepousniotou et al. (2012) investigated the difference between the two types of polysemy – metonymy and metaphor – and homonyms. After a short (50 ms) delay following an ambiguous prime, target words related to any sense of the metonymic polysemous word elicited a reduced N400 effect (a negative deflection that peaks around 400 ms post-stimulus and is associated with semantic processing; Kutas and Hillyard, 1980; for a review, see Kutas and Federmeier, 2011) as compared to the unrelated stimuli. In contrast, for both metaphors and homonyms, the priming effect was more robust for the dominant sense/meaning, although these effects of dominance had different scalp distribution. These results supported the idea that the representation of metaphors in the mental lexicon differs from representations of metonymies and homonyms and involves different neural mechanisms as evidenced by the distinct lateralization of the effects. Using the same paradigm, Meade and Coch (2017) showed a more prominent N400 priming effect for targets related to the dominant meanings of homonyms relative to subordinate meanings, replicating Klepousniotou et al. (2012) findings. They also analyzed modulation of the P600 potential and observed lower P600 amplitude for targets associated with homonyms relative to the unrelated condition. The effect was more prominent for dominant-related targets. The additional analysis of the P600 potential (a positive deflection that peaks around 600 ms post-stimulus) showed lower P600 amplitude for targets associated with homonyms relative to the unrelated condition that was more prominent for dominant-related targets. The P600, which is traditionally associated with syntactic processing and reanalysis (Osterhout and Holcomb, 1992; Kaan et al., 2000), may also reflect integration and pragmatic processing at the discourse level (e.g., interpretation of irony: Regel et al., 2011; “semantic illusion” sentences: Brouwer et al., 2012; and presupposition processing: Domaneschi et al., 2018). The authors interpreted this effect as a marker of the post-lexical reprocessing that reflects integration of the target with the prime.

To get more insight about the time course of sense/meaning activation for ambiguous words, MacGregor et al. (2015) used a longer (750 ms) interstimulus interval. They found that both metonymies and metaphors showed a reduced N400 for targets related to their dominant and subordinate senses, but no such effect was observed for homonyms. Those results indicate that, after a long delay, senses of polysemes remain active, strengthen a unified representation, and facilitate processing of the target, whereas meanings of homonyms might decay due to a lack of supportive context or competition between the unrelated meanings. Targets related to both dominant and subordinate senses of metonymies also showed a reduction of the P600 amplitude relative to unrelated controls. In contrast, metaphors and homonyms showed higher P600 amplitude for subordinate-relative to dominant-related targets. The P600 effect was also observed for targets related to homonyms as compared to the unrelated condition and might reflect difficulties in relating the target to the prime and competition processes.

Bambini et al. (2016) addressed the effects of linguistic context on metaphor processing using minimal and supportive contexts. In the minimal context condition, the authors presented a priming sentence where only one noun was semantically associated with the following target appearing in metaphorical or literal sense (e.g., *Do you know what that fish/lawyer is? A shark*). The results showed an N400-P600 response to the metaphorical compared to the literal condition. In the supportive context, the priming sentence included an additional adjective serving as a unifying feature between the prime and the target (*That fish/lawyer is really aggressive. It is a shark.*), that elicited only a P600 effect. The authors argue that the N400 suppression suggests contextual effects on pragmatic processing, influencing lexical access and retrieval. In contrast, the P600 response is related to the later stage of pragmatic interpretation associated with deriving the metaphorical sense.

Furthermore, the MEG study by Pylkkänen et al. (2006) investigated whether senses/meanings of ambiguous words are stored as the same or different lexical entries. The authors analyzed the interaction between senses/meanings that were activated by the context. The materials included two-word phrases that were preceded either by a prime phrase with an inconsistent sense/meaning of the target word (noun; e.g., *lined paper – liberal paper; river bank – savings bank*) or an unrelated prime (e.g., *military forces – liberal paper; salty dish – savings bank*). Similarly to targets following semantically related phrases (e.g., *liberal paper – daily magazine*), polysemes preceded by primes with an inconsistent sense of the same word elicited an earlier M350 response in the left hemisphere as compared to the control condition. According to the authors, the earlier latency of the M350 effect indicates that different senses of polysemes share one lexical entry. However, in some participants, polysemous targets also elicited a delay in the right-lateralized M350 effect, in contrast to the priming effect observed for semantically related targets. These results show that semantically related representations may interact in the right hemisphere differently depending on whether they belong to the same lexical entry (competition effect) or not (priming effect). In contrast to polysemes, homonymous targets elicited an M350 delay in the left hemisphere as compared to the control condition. The authors suggested that different meanings of a homonym have separate lexical entries and inhibit each other when competing for activation.

Finally, Weiland et al. (2014) contrasted the two types of polysemes – with respect to the role of the literal sense in their processing – using cross-modal masked priming. They analyzed ERPs accompanying the processing of producer-for-product metonymies and metaphors as compared to their literal sense in a sentence context. The polysemous words were either unprimed or preceded by an adjective reflecting a discriminative property of their literal sense. Whereas experimental sentences were presented auditorily, the prime appeared on the screen for 67 and 100 ms before the onset of the target word. The results showed that, in the unprimed condition, metonymies (e.g., *At that time the student read Böll during an assembly*) elicited an N400 effect as compared to the same words with the literal sense (*At that time, the student met Böll during a protest*). Processing metaphors (e.g., *These lobbyists are hyenas, if you believe the kindergarten teacher*) was characterized by a biphasic N400-P600 response as compared to the literal condition (*These carnivores are hyenas, if you believe the kindergarten teacher*) that was related to lexical access and pragmatic aspects of semantic processing. After a prime related to the literal sense (*talented* and *furry* for the metonymy and metaphor examples, respectively), the processing of metonymies did not differ from the control condition, whereas the processing of metaphors was accompanied by a reduced N400 effect and a P600 effect with a later latency. The results suggest that the processing of unprimed metaphors is more costly as compared to metonymies. In addition, pre-activation of the semantic network of the target by a property of the literal sense facilitates the processing of both types of non-literal senses. According to the authors, these results provide evidence for the early activation of literal sense aspects during comprehension of metonymies and metaphors, irrespective of whether it is contextually relevant or not.

The behavioral, eye-tracking, and neuroimaging findings outlined above provide some evidence that ambiguous words are stored and processed differently depending on the kind of relatedness between their senses or meanings. However, previous studies do not allow us to clearly discriminate metonymies, metaphors, and homonyms as constituents of an ambiguity continuum. The two studies shedding the most light on that research question were not designed to pinpoint this specific question, and thus are not fully informative. Pylkkänen et al. (2006), who analyzed the interaction between different senses/meanings of polysemes and homonyms based on the priming effect between them, in the control condition used phrases that were semantically unrelated to the target and included different nouns as a prime. Thus, the effect of semantic relatedness between senses/meanings of ambiguous words relative to the control condition could be contaminated with the effect of (mis)matching between prime and target nouns. In addition, they compared polysemes and homonyms and did not address the difference within polysemy – between metonymies and metaphors. In turn, Weiland et al. (2014) did not analyze the processing of homonyms but showed that metonymies and metaphors are processed differently in a sentence context and that early stages of their comprehension are affected by literal sense aspects. Thus, there is no neurophysiological study available which focuses on all three degrees of semantic relatedness between senses/meanings represented by metonymies, metaphors, and homonyms.

The goal of the present study was to investigate the interaction between different senses/meanings of ambiguous words in a complete design with metonymies, metaphors, and homonyms using ERPs and a priming paradigm. We also improved Klepousniotou et al. (2008) methodology and compared both figurative senses of polysemes with respect to their priming on the literal one within a single word. We analyzed the priming effect that polysemes with metonymic and metaphorical senses exert on their literal sense relative to the control condition in which both the prime and target had the same literal sense. Similarly, for homonyms, the prime meaning either differed from the target one or not. Based on previous research (Klepousniotou et al., 2012; Weiland et al., 2014; MacGregor et al., 2015; Meade and Coch, 2017), we predicted that the priming of ambiguous words by phrases with metonymic or metaphorical senses (for polysemes) and inconsistent meanings (for homonyms) would be more reduced as we move from the metonymic, through the metaphorical, to the homonymous condition, reflected in higher N400 and P600 amplitudes.

## Materials and Methods

### Participants

Thirty native speakers of Russian (21 females, mean age = 24, age range = 18–37 years) participated in the experiment. All participants were right-handed, had normal or corrected to normal vision, no history of neurological diseases, and signed an informed consent in accordance with the Declaration of Helsinki.

### Materials

Experimental materials included 63 polysemous and 63 homonymous nouns. Each polysemous noun had literal, metonymic, and metaphorical senses. Polysemous nouns were selected based on the Active Dictionary of Russian (Apresjan, 2014; Apresjan et al., 2017) and the Great Explanatory Dictionary of the Russian Language (Kuznetsov, 2014) and the theoretical description of polysemy in Russian (Apresjan, 1974). Although dictionary senses may not perfectly reflect word senses in comprehenders’ mental lexicon (Lin and Ahrens, 2000), we believe that dictionary senses from the Active Dictionary of Russian (that aims to facilitate text generation and relies on text corpora) should correspond to word senses that the participants have in mind. Metonymic senses as well as metaphorical senses were derived from literal senses, and there were no derivational relations between metonymic and metaphorical senses. As homonymy and polysemy are sometimes hard to discern, we selected homonymous nouns based on Kachurin (2014) and included in the final set only nouns that were described as homonymous in at least three out of four explanatory dictionaries of Russian.

Although one meaning is usually more frequent (dominant) than others (subordinate) in homonyms, we balanced dominant and subordinate meanings of the used homonyms between the prime and the target, so that they did not differ in their mean frequency [primes: 41% (*SD* = 22), targets: 32% (*SD* = 23), Wilcoxon test: *Z* = 1.4, *p* = 0.18]. Meaning frequency was obtained from Panchenko et al. (2018), where it was estimated based on contexts from the Russian National Corpus^1^. We did not balance sense frequencies of polysemous nouns because it was impossible to find enough nouns with dominant literal, metonymic, or metaphorical senses. Based on the available information from the sense frequency database (http://sensefreq.ruslang.ru/; Lopukhina et al., 2016), we have checked that in 28 out of 63 nouns in our stimuli, the most frequent sense is the literal sense in 22 nouns, the metaphorical sense in three nouns, and the metonymic sense in three nouns. We hypothesize that literal senses are more frequent than non-literal senses in most of our polysemous stimuli. Furthermore, previous studies on sense frequency estimation for Russian nouns showed that, overall, literal senses were the most frequent senses in about 67% of cases (http://sensefreq.ruslang.ru/; Lopukhina et al., 2016).

All nouns were embedded in two-word phrases together with adjectives that highlighted a sense of the polysemous nouns or one meaning of the homonyms. All adjectives were selected from the Active Dictionary of Russian (Apresjan, 2014; Apresjan et al., 2017), the Great Explanatory Dictionary of the Russian Language (Kuznetsov, 2014), or the Russian National Corpus^2^. Additionally, five professional lexicographers from the group of Jury Apresjan, which works on the Active Dictionary of Russian, checked that each adjective corresponds with exactly one sense/meaning of the ambiguous word.

Two-word phrases with literal senses of the polysemous nouns served as targets (for examples, see Table 1 and Supplementary Material). They were preceded by prime phrases with either metonymic or metaphorical sense of the noun. The ERP response to these conditions was compared to the control condition in which targets were preceded by phrases with the same literal sense. Similarly, phrases with homonyms were preceded by phrases with either consistent or inconsistent meanings. Adjectives in the noun phrases did not differ across conditions in frequency [Kruskal-Wallis test: *χ*^2^(2) = 6.88, *p* = 0.16 for polysemes; Mann-Whitney test: *U* = 797, *p* = 0.47 for homonyms] and length in syllables [Kruskal-Wallis test: *χ*^2^(2) = 6.51, *p* = 0.08 for polysemes; Mann-Whitney test: *U* = 1,823, *p* = 0.39 for homonyms]^3^.

**Table 1:** Design of stimuli.

	Condition	Prime	Target
Polysemous word	Metonymic	*zharenyj zajac* “fried hare”	*seryj zajac* “gray hare”
	Metaphorical	*oshtrafovannyj zajac* “fined fare jumper”	
	Literal	*truslivyj zajac* “fearful hare”	
Homonymous word	Inconsistent	*smeshchennyj fokus* “shifted focus”	*tsyrkovoj fokus* “circus trick”
	Consistent	*kartochnyj fokus* “card trick”	
Filler		*jaichnyj belok* “egg white”	^*^geograficheskij belok “geographic white”
		^*^tsifrovoj sindrom “digital syndrome”	^*^iskrennij pozhar “sincere fire”

Meaningless phrases are marked with an asterisk.

The experimental prime-target pairs were split into three experimental lists, so that each participant was presented with 21 trials in each condition and targets did not repeat within a list. One hundred sixty filler pairs of phrases with either the same or different nouns were added to each list: 110 out of 320 phrases were sensible, whereas 210 of them did not make sense (see Table 1 for examples). The order of trials was pseudorandomized within each experimental list, with 10 participants assigned to each of the three lists.

### Procedure

Word phrases within prime-target pairs were presented visually in white on a black background. Each phrase started with a fixation cross (500 ms), followed by an adjective (700 ms) and a noun (until the button press). Participants were asked to judge whether the phrase made sense or not by pressing the left (for “yes”) or right (for “no”) arrow button on the keyboard. The experiment was preceded by a short practice session and lasted about 30 min with a short break in the middle.

### EEG Recording and Analysis

One hundred twenty eight high-impedance ActiCap active electrodes (Brain Products Gmbh, Germany) mounted on an elastic cap and positioned according to the international 10–20 systems were used for electroencephalogram (EEG) data acquisition. The EEG signal was recorded using PyCorder software (Brain Products Gmbh, Germany) with a 500 Hz sampling rate, referenced online to the linked mastoids, and filtered with a 70 Hz low-pass filter. The ground electrode was placed at Fpz, and impedances were kept below 10 kΩ. The EEG signal was processed using the Brain Analyzer software (Brain Products GmbH, Germany). The offline band-pass filter was set at a frequency range of 0.01–40 Hz. Continuous data were then segmented according to experimental conditions with 200 ms before and 1,000 ms after the target noun onset and a DC detrending algorithm was applied. After correction for eye blinks (registered at Fp1) using the Gratton and Coles algorithm (Gratton et al., 1983), artifacts were detected using the individual channel mode and the following criteria: the maximum and minimum values were 150 and −150 μV, respectively; the maximum allowed voltage step between two sample points was 20 μV; and the minimum value difference over an interval of 100 ms was 0.1 μV. Trials containing more than 20% of bad channels were excluded from the analysis. On average, 5% of the data for polysemes and 4% of the data for homonyms were rejected per participant. The baseline correction was performed relative to the 0–200 ms post-stimulus interval. This baseline was defined based on the assumption that the early components of the target noun processing would not differ across conditions. In contrast, the standard pre-stimulus baseline seems to be less appropriate since the difference between the experimental conditions could be potentially observed earlier – on the preceding adjective. The ERPs were calculated according to the experimental conditions.

### Statistics

The effect of priming was examined in the 300–500 ms time windows for the N400 effect and in the 500–800 and 800–1,000 ms time windows for the P600 effect. The later time window was included in the analysis based on results from Weiland et al. (2014) showing a late positivity response (700–900 ms) for the processing of metaphors. For the statistical analysis, the midline electrodes were divided into three groups: frontal (AFz and Fz), central (FCz, Cz, and CPz), and occipital (Pz, POz, and Oz). Six groups of lateral electrodes were created [frontal: left (AF3, AFF1h, AFF5h, F1, F3, F5, FFC1h, FFC3h, FFC5h) and right (AF4, AFF2h, AFF6h, F2, F4, F6, FFC2h, FFC4h, FFC6h); central: left (FC1, FC3, FC5, FCC1h, FCC3h, FCC5h, C1, C3, C5, CCP1h, CCP3h, CCP5h, CP1, CP3, CP5) and right (FC2, FC4, FC6, FCC2h, FCC4h, FCC6h, C2, C4, C6, CCP2h, CCP4h, CCP6h, CP2, CP4, CP6); and occipital: left (CPP1h, CPP3h, CPP5h, P1, P3, P5, PPO1h, PPO5h, PO3, PO7, POO1, O1) and right (CPP2h, CPP4h, CPP6, P2, P4, P6, PPO2h, PPO6h, PO4, PO8, POO2, O2)]. The group values were calculated as an average of the electrodes included.

Event-related potential effects were analyzed using repeated measures ANOVAs separately for polysemous and homonymous words with *Condition* (literal vs. metonymic vs. metaphorical sense prime for polysemous words; and inconsistent vs. consistent prime for homonyms), *Posteriority* (frontal, central, and occipital), and *Hemisphere* (for lateral groups only: left and right) as within-subject factors. When the assumption of sphericity was violated, the Greenhouse-Geisser correction was applied. The values of *p* were adjusted using Bonferroni correction for planned contrasts (literal vs. metonymic and literal vs. metaphorical sense prime; values of *p* were multiplied by two) and *post-hoc* comparisons (values of *p* were multiplied by three, which is the number of levels of the *Posteriority* factor).

## Results

### Behavioral Results

Response accuracy and reaction times for targets in the different experimental conditions are presented in Table 2.

**Table 2:** Response accuracy and reaction times per condition.

	Condition	Accuracy (%): Mean (SD)	Reaction Times (ms): Mean (SD)
Polysemous word	Metonymic	93.3 (8.4)	913 (398)
	Metaphorical	93.3 (9.6)	874 (284)
	Literal	95.4 (6.2)	825 (255)
Homonymous word	Inconsistent	78.6 (13.2)	947 (277)
	Consistent	87.1 (10.2)	923 (320)

According to the statistical analysis of accuracy (Wilcoxon test for metonymic vs. literal and metaphorical vs. literal conditions with a Bonferroni correction of values of *p*), polysemous targets with metonymic and metaphorical primes did not differ from the control condition (*p*s > 0.05). In contrast, the difference between homonymous targets with consistent and inconsistent primes was statistically significant (Wilcoxon test: *Z* = 46, *p* = 0.006): participants made more errors with homonymous targets preceded by inconsistent primes as compared to the control condition.

The analysis of reaction times for polysemous (Wilcoxon test for metonymic vs. literal and metaphorical vs. literal conditions with a Bonferroni correction of values of *p*) and homonymous (*t*-test for inconsistent vs. consistent condition) targets did not show significant difference for any of the contrasts (*p*s > 0.05).

### ERP Results

**Figure 1:**
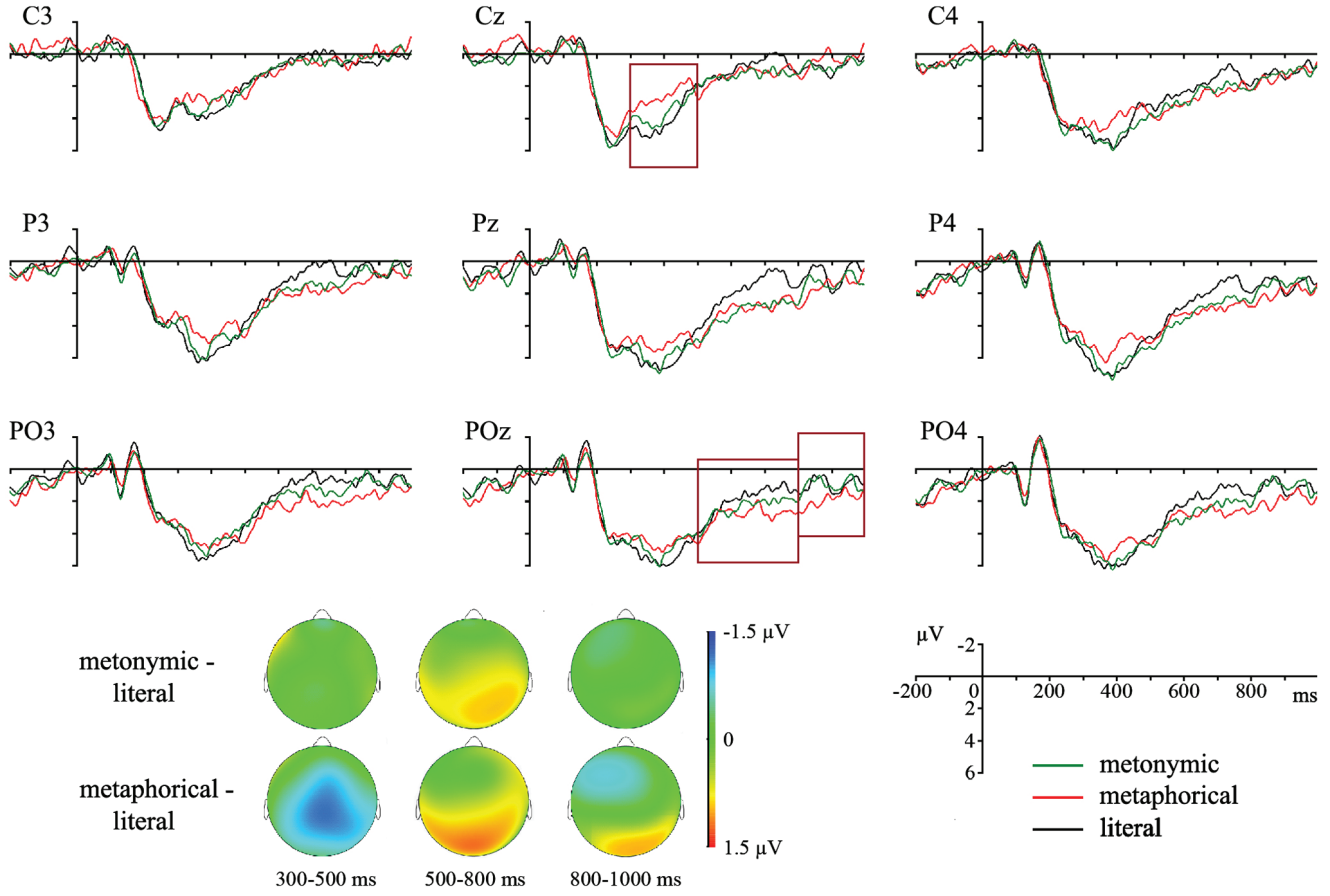
Grand average ERPs for polysemous words preceded by primes with metonymic (green line), metaphorical (red line), and literal (black line) senses, and topographic scalp distribution of the amplitude differences. The N400 and P600 time windows are highlighted. Negative is plotted up.

In the 300–500 ms time window, statistical analysis of the ERP response to polysemous targets with three levels of *Condition* showed a marginally significant effect of *Condition* [*F*(2,58) = 2.89, *p* = 0.063, *η*^2^ = 0.091] in the midline electrode groups with no significant interaction with the *Posteriority* factor. Planned contrasts revealed a significant difference in the N400 amplitude between the metaphorical and control conditions [*F*(1,29) = 7.01, *p* = 0.026, *η*^2^ = 0.195, mean difference 0.78 μV; see Figure 1]. No significant effect of *Condition* or its interaction with other factors was observed in the lateral electrode groups.

Analysis of the ERPs accompanying the processing of homonymous targets did not show any significant difference between the two experimental conditions in the midline or lateral electrode groups.

In the 500–800 ms time window, statistical analysis of the ERPs elicited by polysemous targets in the three experimental conditions did not show significant effect of *Condition* or its interaction with the *Posteriority* factor in the midline electrode groups. In the lateral electrode groups, a marginally significant effect of *Condition* [*F*(2,58) = 2.93, *p* = 0.070, *η*^2^ = 0.173] was observed without significant interaction with other factors. Planned contrasts showed that the processing of polysemous targets preceded by metaphorical phrases is characterized by a significant P600 effect [*F*(1,29) = 6.04, *p* = 0.040, *η*^2^ = 0.172, mean difference 0.658 μV] as compared to the control condition.

**Figure 2:**
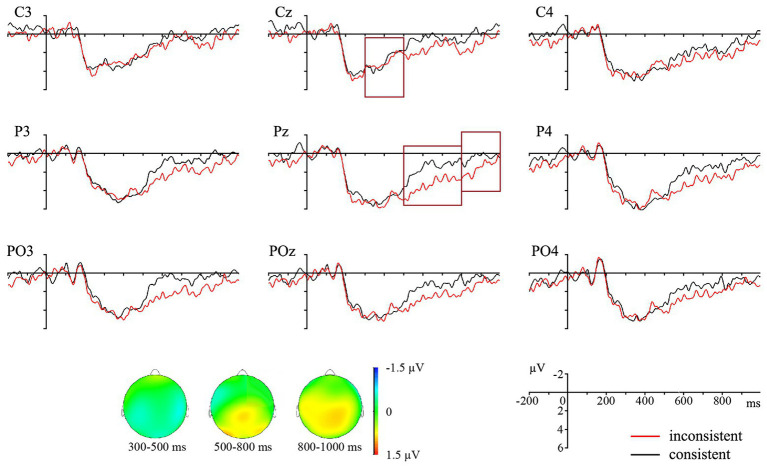
Grand average ERPs for homonymous words preceded by primes with inconsistent (red line) and consistent (black line) meanings and topographic scalp distribution of the amplitude differences. The N400 and P600 time windows are highlighted. Negative is plotted up.

The difference between the inconsistent and consistent conditions for homonymous targets was reflected in a marginally significant effect of *Condition* [*F*(1,29) = 3.94, *p* = 0.057, *η*^2^ = 0.120, mean difference 0.892 μV] and a marginally significant *Condition* by *Posteriority* [*F*(2,58) = 2.99, *p* = 0.058, *η*^2^ = 0.093] interaction in the midline electrode groups (see Figure 2). According to *post-hoc* ANOVAs, the P600 effect that accompanies the processing of homonymous targets preceded by inconsistent primes as compared to the control condition reaches significance [*F*(1,29) = 7.21, *p* = 0.036, *η*^2^ = 0.199, mean difference 1.323 μV] in the occipital electrode group. In the lateral electrode groups, a significant *Condition* by *Posteriority* [*F*(2,58) = 5.22, *p* = 0.015, *η*^2^ = 0.152] interaction was observed. *Post-hoc* analysis showed a marginally significant difference between the two experimental conditions [*F*(1,29) = 5.86, *p* = 0.066, *η*^2^ = 0.168, mean difference 1.010 μV] in the occipital electrode groups.

In the 800–1,000 ms time window, statistical analysis for polysemous targets with three levels of *Condition* showed a significant *Condition* by *Posteriority* [*F*(4,116) = 3.89, *p* = 0.015, *η*^2^ = 0.118] interaction in the midline electrode groups. Further analysis did not show significant difference across conditions at any level of *Posteriority*. Similarly to the midline electrode groups, statistical analysis of the ERPs elicited by polysemous targets in the three experimental conditions revealed a significant *Condition* by *Posteriority* [*F*(4,116) = 6.26, *p* = 0.003, *η*^2^ = 0.117] interaction in the lateral electrode groups. *Post-hoc* ANOVAs did not show significant effect of *Condition* or its interaction with the *Hemisphere* factor at any level of *Posteriority*.

The difference between homonymous targets with inconsistent and consistent primes was reflected in a marginally significant effect of *Condition* [*F*(1,29) = 4.00, *p* = 0.055, *η*^2^ = 0.121, mean difference 0.72 μV] in the midline electrode groups with no significant interaction with the *Posteriority* factor. Statistical analysis in the lateral electrode groups showed a marginally significant effect of *Condition* [*F*(1,29) = 3.25, *p* = 0.082, *η*^2^ = 0.101, mean difference 0.892 μV] and a marginally significant *Condition* by *Posteriority* [*F*(2,58) = 2.49, *p* = 0.092, *η*^2^ = 0.079] interaction. *Post-hoc* ANOVAs did not reveal a significant effect of *Condition* or its interaction with the *Hemisphere* factor at any level of *Posteriority*.

## Discussion

We investigated the interaction between senses/meanings of Russian ambiguous words based on an analysis of ERPs characterizing the priming effect among phrases with different degrees of semantic relatedness – metonymies, metaphors, and homonyms. Our behavioral data did not reveal any significant difference in accuracy and reaction times among the target polysemes with a literal sense preceded by primes with metonymic, metaphorical, and the literal sense itself. According to these results, switching between the metonymic or metaphorical sense to the literal sense of polysemes did not imply involvement of additional processing resources. Our results differ from the results of behavioral experiments on processing ambiguous words without any preceding context (Klepousniotou and Baum, 2007, in the auditory modality) and after a semantically associated sentence (Klepousniotou, 2002) that demonstrated a difference in processing metonymies and metaphors. Moreover, the results of Klepousniotou et al. (2008) experiment with a similar design showed that the difference between conditions with consistent and inconsistent primes was more pronounced for metaphors as compared to metonymies. The discrepancy between our and Klepousniotou et al. results may be related to the difference in presentation mode used in the two experiments: in contrast to Klepousniotou et al. (2008) who presented the two-word phrases as a whole, during our experiment participants saw adjectives for a limited amount of time. Our behavioral data characterize processing of the target noun only, which could result in a lower sensitivity of these measures. In addition, in our design, polysemes in the target phrase always had a literal sense that could facilitate the integration process. Thus, our behavioral results were unable to distinguish metonymies and metaphors with respect to the relatedness between their senses.

In contrast to the behavioral measures, our electrophysiological data demonstrate the difference between metonymic and metaphorical senses of the polysemes in the amount of their priming for the literal sense. Concerning metonymy, our ERP analysis did not reveal any significant difference in N400 or P600 amplitudes between target phrases with a literal sense preceded by primes with the literal sense itself or the metonymic sense. These results indicate that the priming effect is comparable for phrases with metonymic and literal senses. Our results are in line with Weiland et al. (2014) data on processing polysemes with figurative senses as compared to a literal sense in sentential context: the sentences were either primed with a word associated with the literal sense or not. It was demonstrated that the N400 effect characterizing processing metonymies in the unprimed condition can be eliminated when they are preceded by a prime that is semantically related to the literal sense. Similar results were observed in the experiments of Klepousniotou et al. (2012), as well as of MacGregor et al. (2015), on processing ambiguous words without preceding context. The authors showed that metonymies can provide a comparable priming effect on targets that are semantically related to their metonymic or literal sense. Thus, the priming effect for metonymic and literal senses of polysemes, evidenced in our study by similar electrophysiological signatures, supports the suggestion that these senses share a single representation in the mental lexicon, with spreading activation between them.

In contrast to metonymic primes, significant N400 and P600 effects were observed for target words with literal senses preceded by phrases with metaphorical senses as compared to the control condition. The P600 effect had a form of a long shift and reached significance in both 500–800 and 800–1,000 ms time windows, with a wider spatial distribution in the earlier time window. These effects show that metaphorical senses of polysemous words have a very limited priming effect on literal senses of the same words. Following Bambini et al. (2016) view, we can relate the N400 effect to pragmatic aspects of lexical access and retrieval, whereas the P600 response may reflect a later stage of interpretation in order to activate the intended sense. In addition, the P600 amplitude may be modulated by relative difficulties that participants had with the integration of a different sense into the context and performing a sensicality judgment that followed the target word processing. These results support the idea that the semantic relatedness between a word’s literal and metaphorical senses is less prominent as compared to their metonymic senses. The difference in the processing of metonymies and metaphors is in line with the results of previous electrophysiological studies, showing that metonymic and metaphorical primes can differently modulate the N400 and P600 potentials that accompany the processing of targets related to their dominant and subordinate senses (Klepousniotou et al., 2012; MacGregor et al., 2015).

Our results are consistent with the response observed for polysemes with a metaphorical sense primed by a word associated with their literal sense in the study by Weiland et al. (2014). They also showed that for both primed metaphors and metonymies, the N400 effect was lower as compared to the unprimed condition. Based on these priming effects, they argued that, independent of the context, the literal sense is activated at early stages of processing polysemes with a metonymic or metaphorical sense. According to the authors, these results are in line with the indirect access account indicating that the metaphorical sense is always activated *via* the literal one. For example, the Relevance theory by Carston (2010) predicts a pragmatical adjustment of the literal concept in order to create a non-literal interpretation. Similarly, the blending theory claims that a figurative sense is constructed from a blending space based on both source (literal sense) and target (metaphorical sense) domains (Fauconnier and Turner, 2002; Coulson and Oakley, 2005). However, we can also suggest that the facilitation effect observed in Weiland et al. study may result from the activation of the literal sense by the prime that could spread over the metonymic or metaphorical sense semantically related to the literal one. According to this view, these results do not necessarily support the idea that the activation of non-literal senses of polysemes (unprimed by the literal one) is preceded by activation of the literal sense. They may actually be compatible with the direct access account that negates the additional step in the processing of metaphors (e.g., Glucksberg, 2008). The very limited priming of polysemes with the literal sense by phrases with the metaphorical sense observed in our study provides support for the direct access hypothesis, since the literal sense of metaphors was not activated when the prime was related to the metaphorical sense. However, another explanation is also possible: the literal sense of the prime activated at early stage could decay after accessing the metaphorical one. Although our results do not allow us to disentangle between these two theoretical accounts, they indicate that after accessing the metaphorical sense the literal one remains or has low activation.

Unlike our behavioral data for polysemes, which did not show differences across conditions, accuracy rates for homonymous targets were lower for phrases preceded by primes with the inconsistent meaning as compared to the consistent condition: participants made more errors during sensicality judgments when the prime and target phrases activated different meanings of a homonym. The difference between conditions in the behavioral measures indicate that the effect of meaning consistency between the prime and target for homonyms was more pronounced as compared to the effect of consistency between senses for polysemes. These results are also in line with the results of the experiment of Klepousniotou et al. (2008) that showed lower accuracy for homonyms with a subordinate meaning following an inconsistent prime.

The difference between the two experimental conditions with homonyms is also reflected in the electrophysiological response: a P600 effect was observed for phrases with homonyms preceded by the prime with a different meaning relative to the control condition. Similar to the effect accompanying processing of polysemous words preceded by phrases with metaphorical senses, this effect had a long duration; the difference between the two experimental conditions was found in both 500–800 and 800–1,000 ms time windows with a wider spatial distribution in the later time window. Surprisingly, the two experimental conditions did not differ regarding the N400 amplitude. In contrast to our results, some previous studies reported modulation of the N400 effect for targets related to homonymous primes presented after a short (50 ms) interstimulus interval and could be associated with automatic activation of word meanings (Klepousniotou et al., 2012; Meade and Coch, 2017). The absence of the N400 effect in our study corresponds to the results of MacGregor et al. (2015) experiment showing no N400 reduction for targets related to any meaning of homonymous primes and presented after a long (750 ms) interstimulus interval as compared to unrelated controls. The authors argue that, after a long delay, competing meanings of homonyms were no longer active in such a limited context. In our experiment, the pause between prime and target homonymous nouns consisted of 500 ms cross and 700 ms adjective presentations that could be enough for the activated meaning to decay. However, the semantically related adjective preceding the target noun could provide support for this meaning. Moreover, if the activated meaning in the control condition also decayed, we would not expect any later effects, since our target homonymous phrases with a primed meaning of the homonym did not differ across conditions.

Lexical access to the target meaning of homonyms preceded by an inconsistent prime may be inhibited by the activated meaning that could result in the absence of the N400 effect. According to Pylkkänen et al. (2006) MEG results, the M350 response (analog of the N400 potential) to homonyms preceded by the same word with an inconsistent meaning had later latency as compared to the condition with unrelated primes. In contrast to semantically related prime-target pairs with different nouns and polysemes, which showed a significant priming effect, an inhibitory effect was observed for homonymous targets with the inconsistent meaning of the prime as compared to unrelated primes. This effect may be even more prominent when target phrases in the control condition are primed by the same meaning of the homonym as in our experiment. Thus, the absence of a significant difference in the N400 amplitude between the two conditions might be caused by delayed access to the target meaning following the inconsistent prime.

In contrast to the idea that, in both conditions, the activated meanings of homonyms decay before presentation of the target noun, we found an increase in the P600 amplitude for targets preceded by an inconsistent prime as compared to the control condition. Similarly, MacGregor et al. (2015) reported a P600 effect for targets related to one of the meanings of homonymous primes as compared to unrelated controls. According to them, the low relatedness between the meanings of homonyms was supposed to prevent them from collaboration and lead to the competition that resulted in the P600 effect characterizing the processing of the target. It is important to note that, in the experiment of MacGregor et al. (2015), homonyms were used as a prime, whereas in our experiment their target meaning was biased by a preceding adjective. Despite this, the target meaning seems not to be activated in the standard N400 time window after an inconsistent prime. Since no N400 effect with a later latency was observed, we can suppose that the P600 effect might reflect access to the target meaning in the inconsistent condition. Following MacGregor et al. (2015) assumption, we can also suppose a further resolution of the conflict between the two meanings of the homonym that is followed by integration of the target meaning into the context and a sensicality judgment. Our results are in line with the previous studies, showing that the P600 effect may be an index of semantic and pragmatic processing (Regel et al., 2011; Brouwer et al., 2012; Bambini et al., 2016). The later access to the target meanings of homonyms preceded by an inconsistent prime and further process might be reflected by the lower accuracy observed for this condition relative to the control one.

The observed ERP results are evidence for discrepancy in comprehension and interaction between the senses/meanings of different types of ambiguous words – metonymies, metaphors, and homonyms. Similarly to metonymies, the inconsistency between priming and target meanings of the homonyms did not induce modulation of the N400 amplitude. However, we suggest that the absence of the N400 as well as P600 effects during comprehension of metonymies is caused by comparable priming of the literal sense by phrases with the literal and metonymic senses. Concerning homonyms, the similar N400 amplitude in the two experimental conditions is supposed to reflect inhibition of lexical access to the target meaning of the noun following an inconsistent prime. In contrast to metonymies and homonyms, the processing of metaphors with the literal sense preceded by primes with the metaphorical sense elicited an N400 effect accompanying lexical access to the target sense that is semantically related to the prime. In addition, the inconsistency between priming and target senses of metaphors resulted in a P600 effect that could be a marker of competition, integration of the target sense into the context and sensicality judgment. The P600 effect characterizing the processing of homonyms in the inconsistent condition might also reflect processes of reanalysis and activation of a different meaning.

Our study has several limitations. The first limitation lies in the number of stimuli (21 per condition) used in the experiment, which is lower than usual in ERP studies. This restriction is related to the low number of Russian polysemes that have both metonymic and metaphorical senses. The second limitation is the incomplete information about sense frequencies of polysemous nouns. We had no information about sense frequencies for most of our stimuli (35 out of 63). However, Lopukhina et al. (2016) showed that literal senses are usually the most frequent senses in Russian nouns. Accordingly, we can suppose that, in the majority of our stimuli, primes with both metonymic and metaphorical senses were followed by targets with the most frequent literal sense. Based on this, we presume that the observed difference in the processing of target nouns preceded by the metonymic and metaphorical primes cannot be explained solely by the sense frequencies. The third limitation lies in the difference in collocational strength between prime phrases with literal and metonymic senses of polysemous words. Although the collocational strength in phrases with metonymic senses was lower as compared to phrases with literal senses, there was no difference in the ERP response between the target nouns with a literal sense preceded by primes with the literal sense itself or metonymic sense. Importantly, in our stimuli, there was no difference in collocational strength between literal and metaphorical phrases. Thus, we suppose that the N400 and P600 effects elicited by target words with literal senses preceded by phrases with metaphorical senses are not due to the difference in the probability of adjective-noun co-occurrence. The final limitation is that we used a post-stimulus baseline for the target noun in order to eliminate the influence of any possible priming effect on the preceding adjective, although it is rarely applied in the ERP research.

## Conclusions

The present study on comprehension of ambiguous words (nouns) provides additional evidence for the difference in mechanisms underlying the processing of polysemous and homonymous words and interactions between their senses/meanings. In addition, an analysis of the interplay between the literal, metonymic, and metaphorical senses of polysemous words allowed us to contrast metonymic and metaphorical relations within polysemy. Our results confirm both the difference in processing between polysemes and homonyms and the discrepancy between the two types of polysemy – metonymy and metaphor. According to the results, literal and metonymic senses are close to each other and might share a single mental representation. In contrast, the distance between literal and metaphorical senses is more prominent, which is reflected in processing mechanisms partly similar to those involved in the interaction between meanings of homonymous words.

## Data Availability Statement

The raw data supporting the conclusions of this manuscript will be made available by the authors, without undue reservation, to any qualified researcher.

## Ethics Statement

This study was approved by the Committee on Interuniversity Surveys and Ethical Assessment of Empirical Research of the National Research University Higher School of Economics (HSE). All subjects gave written informed consent in accordance with the Declaration of Helsinki.

## Author Contributions

Conception and design of the experiment: AL, AY, and OD. Programming of the experiment, data collection, and statistical analysis of experimental results: AY. Preparation of the final document: AY, AL, and OD. All authors contributed to the article and approved the submitted version.

### Conflict of Interest

The authors declare that the research was conducted in the absence of any commercial or financial relationship that could be construed as a potential conflict of interest.

## Footnotes

^1^http://www.ruscorpora.ru/en/

^2^http://www.ruscorpora.ru/en/

^3^Following the reviewers’ suggestions, we measured the degree of adjective-noun collocational strength of the prime and target phrases. For that, we calculated normalized pointwise mutual information that is the association measure between adjectives and nouns in a corpus and is based on the frequencies of each word and the frequency of the noun phrase (Bouma, 2009). We applied the algorithm to the lemmatized state-of-the-art Russian National Corpus (577 million tokens; http://www.ruscorpora.ru/). Wilcoxon test showed that in adjective-noun collocations for polysemous nouns and prime phrases with metonymic senses had lower values than prime phrases with literal (*Z* = −3.5, *p* < 0.003) and metaphorical senses (*Z* = −2.3, *p* = 0.06). There was no significant difference between the literal and metaphorical conditions.
